# Does Perceived Neighborhood Walkability and Safety Mediate the Association Between Education and Meeting Physical Activity Guidelines?

**DOI:** 10.5888/pcd12.140570

**Published:** 2015-04-09

**Authors:** Michael Pratt, Shaoman Yin, Robin Soler, Rashid Njai, Paul Z. Siegel, Youlian Liao

**Affiliations:** Author Affiliations: Robin Soler, Rashid Njai, Paul Z. Siegel, Youlian Liao, National Center for Chronic Disease Prevention and Health Promotion, Centers for Disease Control and Prevention, Atlanta, Georgia; Shaoman Yin, SciMetrika, LLC, Durham, North Carolina.

## Abstract

The role of neighborhood walkability and safety in mediating the association between education and physical activity has not been quantified. We used data from the 2010 and 2012 Communities Putting Prevention to Work Behavioral Risk Factor Surveillance System and structural equation modeling to estimate how much of the effect of education level on physical activity was mediated by perceived neighborhood walkability and safety. Neighborhood walkability accounts for 11.3% and neighborhood safety accounts for 6.8% of the effect. A modest proportion of the important association between education and physical activity is mediated by perceived neighborhood walkability and safety, suggesting that interventions focused on enhancing walkability and safety could reduce the disparity in physical activity associated with education level.

## Objective

Physical activity is an important contributor to health and well-being. People with more education consistently report greater participation in physical activity (1). It is also well established that neighborhood walkability and safety influence participation in physical activity (2,3). However, the mediating role of the neighborhood environment, in particular walkability and safety, on the association between education level and physical activity level has not been quantified. We hypothesized that perceived neighborhood walkability and safety are important mediators of the relationship between education level and physical activity level. If true, this suggests that efforts to make environments more amenable to physical activity in neighborhoods characterized by low education levels may help to reduce disparities in physical activity (4,5).

## Methods

We analyzed aggregated data collected in 2010 and 2012 from 2 modified Behavioral Risk Factor Surveillance System (BRFSS) surveys (6) in the 39 “Communities Putting Prevention to Work” (CPPW) communities (7) that included questions on perceived neighborhood walkability and safety in addition to the usual BRFSS questions on education and physical activity. CPPW was a 2-year initiative that funded 50 communities to implement policy, systems, and environmental interventions to reduce obesity and tobacco consumption (7). The median response rate based on CASRO (Council of American Survey Research Organizations [http://www.casro.org/]) was 55% in 2012 (information not available for all communities in 2010). The final analytic sample consisted of 104,084 adults aged 18 years or older after exclusion of those for whom data were missing. Education was classified into 4 levels (less than high school, high school, some college, and college graduate). Physical activity was dichotomized to meeting or exceeding the 2008 US physical activity guidelines (ie, doing at least 150 minutes per week of moderate-intensity, or 75 minutes per week of vigorous-intensity aerobic physical activity, or an equivalent combination of the two) versus not meeting those guidelines (1). Perceived neighborhood environment for walking was categorized as very pleasant, somewhat pleasant, not very pleasant, and not at all pleasant. Perceived neighborhood safety was categorized as extremely safe, quite safe, slightly safe, and not at all safe. We conducted bivariate analyses to examine the inter-relationship between education level (exposure) and physical activity level (response), education and the mediators (neighborhood walkability and safety), and the mediators and physical activity. Then, we used structural equation modeling (SEM) (8) (Figure) to select the best-fit model and to estimate the direct effect of exposure on response, indirect effect of exposure on response via mediator, and the total effect (sum of direct and indirect effect). We included the following covariates in the SEM model: age (5 age groups), sex (male vs female), race/ethnicity (non-Hispanic white vs any other race), geographic location (urban area/large city vs rural area/small city), and intervention focus of the community (obesity only or obesity and tobacco vs tobacco only). Lastly, we calculated the mediation proportion as the ratio (percentage) of indirect effect over total effect (9). Descriptive and bivariate analyses were conducted using SAS-callable [SUDAAN, version 9.3, Research Triangle Institute] SUDAAN to account for the complex sample design. SEM analysis accounting for complex sampling features was conducted using Mplus, version 6.0 (Muthén and Muthén).

**Figure:**
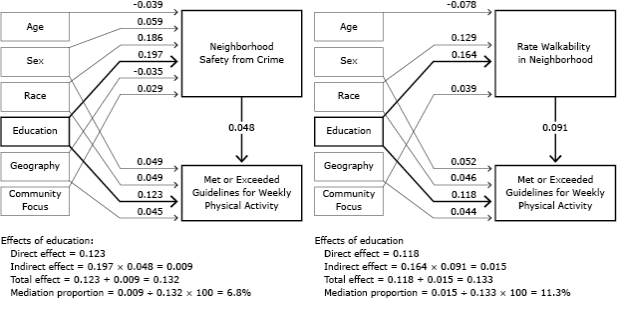
Mediation models from education level to meeting physical activity guidelines showing the direct effects of education, the indirect (mediated) effects acting through perceived neighborhood safety and perceived neighborhood walkability, and the proportion of the overall effect due to mediation, Communities Putting Prevention to Work: Behavioral Risk Factor Surveillance System 2010 and 2012. Numbers in the figure are standardized regression coefficients in the structural equation models. **Table:** 

A pair of flow charts shows the relationships between education level and meeting physical activity guidelines, taking into account the covariates of age, sex, race/ethnicity, geography, and community focus and the mediating effects of perceived neighborhood safety from crime and perceived neighborhood walkability. The influence of education level and each significant covariate on meeting physical activity guidelines and perceived neighborhood safety from crime and perceived neighborhood walkability are reported as standardized regression coefficients from the structural equation models. The effects of perceived neighborhood safety from crime and perceived neighborhood walkability on meeting physical activity guidelines are also reported as standardized regression coefficients from the structural equation models in the following tables.		
**Flow Chart A. Mediation Model From Education to Meeting Physical Activity Guidelines Showing the Direct Effects Of Education and the Indirect (Mediated) Effect Acting Through Perceived Neighborhood Safety^a^**		
**Covariates and Mediating Effects**	**Standardized Regression Coefficients**	
	**Neighborhood Safety**	**Physical Activity Guidelines**

**Age**	**−0.039**	—
**Sex**	**0.059**	**0.049**
**Race**	**0.186**	**0.049**
**Education**	**0.197**	**0.123**
**Geography**	**−0.035**	**0.045**
**Community focus**	**0.029**	—
**Neighborhood safety**	—	**0.048**

**Flow Chart B. Mediation Model From Education to Meeting Physical Activity Guidelines Showing the Direct Effects of Education Level and the Indirect (Mediated) Effect Acting Through Perceived Neighborhood Walkability^b^**		
**Covariates and Mediating Effects**	**Neighborhood Walkability**	**Physical Activity Guidelines**

**Age**	**−0.078**	**0.052**
**Sex**	—	**0.046**
**Race**	**0.129**	**0.118**
**Education**	**0.164**	**0.044**
**Geography**	—	—
**Community focus**	**0.039**	**0.091**
**Neighborhood walkability**	—	—

SEM showed that level of education had significant direct and indirect effects on meeting physical activity guidelines. The indirect effects are partially mediated by both perceived neighborhood walkability and perceived neighborhood safety from crime. Neighborhood walkability mediates 11.3% of the total effect of education on meeting physical activity guidelines. Neighborhood safety mediates 6.8% of the total effect of education on meeting physical activity guidelines.		

Abbreviation: —, not applicable.

^a^ Effects of education level with mediation by perceived neighborhood safety from crime: direct effect = 0.123; indirect effect = 0.197 × 0.048 = 0.009; total effect = 0.123 + 0.009 = 0.132; mediation proportion = 0.009 ÷ 0.132 × 100 = 6.8%.

^b^ Effects of education with mediation by perceived neighborhood walkability: direct effect = 0.118; indirect effect = 0.164 × 0.091 = 0.015; total effect t =0.118 + 0.015 = 0.133; mediation proportion = 0.015 ÷ 0.133 × 100 = 11.3%.

## Results

The weighted overall sample was 51.8% female (95% confidence interval [CI], 50.6%–52.9%), and 53.2% (95% CI, 52.0%–54.3%) met or exceeded the 2008 US guidelines for weekly physical activity. Among respondents 16.9% (95% CI, 15.9%–18.0%) reported less than high school education, 24.2% (95% CI, 23.3%–25.2%) high school, 28.5% (95% CI, 27.5%–29.6%) some college, and 30.3% (95% CI, 29.4%–31.3%) were college graduates. Walking in the neighborhood was rated as very pleasant by 54.7% (95% CI, 53.5%–55.9%), somewhat pleasant by 35.7% (95% CI, 34.6%–36.8%), not very pleasant by 6.8% (95% CI, 6.1%–7.5%), and not at all pleasant by 2.8% (95% CI, 2.5%–3.2%) of respondents. Neighborhood safety from crime was perceived as extremely safe by 20.3% (95% CI, 19.4%–21.1%), quite safe by 50.9% (95% CI, 49.7%–52.0%), slightly safe by 24.0% (95% CI, 23.0%–25.1%), and not at all safe by 4.9% (95% CI, 4.4%–5.4%) of respondents. Bivariate analyses showed that with each increasing level of education the proportion of respondents reporting they met physical activity guidelines increased, and perceived neighborhood walkability and safety was greater (Table). The 2 perceived environmental measures were also associated with level of physical activity. SEM showed that level of education had significant direct and indirect effects on meeting physical activity guidelines (Table) (Figure). The indirect effects are partially mediated by both perceived neighborhood walkability and perceived neighborhood safety from crime. Neighborhood walkability mediates 11.3% of the total effect of education on meeting physical activity guidelines. Neighborhood safety mediates 6.8% of the total effect of education on meeting physical activity guidelines.

**Table:** Bivariate Relationships^a^ and Estimates of Effects Among Education Level, Perceived Neighborhood Environment, and Meeting Physical Activity Guidelines, Communities Putting Prevention to Work Behavioral Risk Factor Surveillance System, 2010 and 2012

Characteristics	Neighborhood Safe from Crime^b^				Rate Walking in Neighborhood^b^				Met or Exceeded Physical Activity Guidelines^c^
	Extremely Safe	Quite Safe	Slightly Safe	Not At All Safe	Very Pleasant	Somewhat Pleasant	Not Very Pleasant	Not At All Pleasant	
**Education level**									
Less than high school	13.6	35.6	41.4	9.4	41.9	40.8	12.2	5.2	42.0
High school	17.7	50.8	25.8	5.7	50.8	39.5	6.4	3.2	50.8
Some college	19.3	53.0	23.1	4.6	54.5	36.3	6.6	2.6	54.8
College graduate	26.9	57.5	13.7	1.8	65.2	29.2	4.2	1.5	59.7
**Neighborhood safe from crime**									
Extremely safe	—	—	—	—	—	—	—	—	57.3
Quite safe	—	—	—	—	—	—	—	—	54.2
Slightly safe	—	—	—	—	—	—	—	—	49.1
Not at all safe	—	—	—	—	—	—	—	—	44.6
**Walking in neighborhood**									
Very pleasant	—	—	—	—	—	—	—	—	56.9
Somewhat pleasant	—	—	—	—	—	—	—	—	49.7
Not very pleasant	—	—	—	—	—	—	—	—	45.5
Not at all pleasant	—	—	—	—	—	—	—	—	43.7
**Estimate of effects**									
Education → outcome, total^d^	0.132 (*P* < .001)				0.133 (*P* < .001)				—
Education → outcome, direct^d^	0.123 (*P* < .001)				0.118 (*P* < .001)				—
Education → mediator → outcome, indirect^d^	0.009 (*P* = .004)				0.015 (*P* < .001)				—
Mediation proportion	6.80				11.30				—

Abbreviation: —, not applicable.

a The Cochran-Mantel-Haenszel trend test was used in the bivariate analysis. All *P* values are less than .001.

b Values are percentages unless otherwise indicated.

c Met or exceeded the US 2008 guidelines for weekly physical activity (1).

d Values are standardized regression coefficients.

## Discussion

Our analysis of the 2010 and 2012 CPPW BRFSS demonstrates that a modest proportion of the important association between education level and meeting physical activity guidelines is mediated by perceived neighborhood walkability and safety. Although the mediating effects are relatively small, these results suggest that interventions focused on enhancing walkability and safety in communities that face challenges in these areas might cut into the consistently observed disparity in meeting physical activity guidelines associated with education level. Given the difficulty of directly intervening on the underlying socioeconomic determinants of health (eg, income, poverty, employment, education), public health strategies focused on improving the environment so that healthy options become more feasible appear promising (4,10). Improving the environment has been the basis for a series of programs supported by the Centers for Disease Control and Prevention (CPPW, Community Transformation Grants, Racial and Ethnic Approaches to Community Health) that provided funds to states and communities for sustainable policy, system, and environmental interventions (7).

Our results are limited by the cross sectional design of the CPPW BRFSS. We cannot infer causality between exposures and outcomes. Although the overall sample was large and similar in key characteristics (including physical activity level [11]) to national samples, our sample was drawn from only 39 communities and is not nationally representative. A strength of the BRFSS and these analyses is the information available on a large number of potential confounders and the ability to control for them in the analyses. Physical activity, education, walkability, and safety were all assessed by self-report and are thus subject to recall and social desirability bias. It is possible that with an objective measure of physical activity, such as by accelerometer and objective measures of the environment, such as GIS-based walkability indices and actual crime reports, the mediating effects of neighborhood walkability and safety on the education–physical activity relationship might be better elucidated. Studies of this type should become a priority. However, despite these limitations our results do suggest that part of the observed association between education level and meeting physical activity guidelines is mediated by the neighborhood environment, and that interventions designed to improve community environments have the potential to close equity-based gaps in physical activity.
